# The treatment efficacy of bone tissue engineering strategy for repairing segmental bone defects under diabetic condition

**DOI:** 10.3389/fbioe.2024.1379679

**Published:** 2024-04-26

**Authors:** Xiangsheng Wang, Can Xiang, Chunhua Huang, Hanxiao Cheng, Zhentao Zhou, Jufang Zhang, Hui Xie

**Affiliations:** ^1^ Department of Plastic Surgery, Jingshan Union Hospital, Union Hospital, Huazhong University of Science and Technology, Hubei, China; ^2^ Department of Plastic Surgery, Affiliated Hangzhou First People's Hospital, Zhejiang University School of Medicine, Hangzhou, Zhejiang, China; ^3^ Department of Research, Union Hospital, Tongji Medical College, Huazhong University of Science and Technology, Wuhan, Hubei, China; ^4^ Department of Geriatrics, Tongji Hospital, Tongji Medical College, Huazhong University of Science and Technology, Wuhan, Hubei, China

**Keywords:** diabetes mellitus, bone tissue engineering, mesenchymal stem cells, decalcified bone matrix, bone regeneration

## Abstract

**Background:**

Diabetes mellitus is a systematic disease which exert detrimental effect on bone tissue. The repair and reconstruction of bone defects in diabetic patients still remain a major clinical challenge. This study aims to investigate the potential of bone tissue engineering approach to improve bone regeneration under diabetic condition.

**Methods:**

In the present study, decalcified bone matrix (DBM) scaffolds were seeded with allogenic fetal bone marrow-derived mesenchymal stem cells (BMSCs) and cultured in osteogenic induction medium to fabricate BMSC/DBM constructs. Then the BMSC/DBM constructs were implanted in both subcutaneous pouches and large femoral bone defects in diabetic (BMSC/DBM in DM group) and non-diabetic rats (BMSC/DBM in non-DM group), cell-free DBM scaffolds were implanted in diabetic rats to serve as the control group (DBM in DM group). X-ray, micro-CT and histological analyses were carried out to evaluate the bone regenerative potential of BMSC/DBM constructs under diabetic condition.

**Results:**

In the rat subcutaneous implantation model, quantitative micro-CT analysis demonstrated that BMSC/DBM in DM group showed impaired bone regeneration activity compared with the BMSC/DBM in non-DM group (bone volume: 46 ± 4.4 mm^3^ vs 58.9 ± 7.15 mm^3^, *p < 0.05). In the rat femoral defect model, X-ray examination demonstrated that bone union was delayed in BMSC/DBM in DM group compared with BMSC/DBM in non-DM group. However, quantitative micro-CT analysis showed that after 6 months of implantation, there was no significant difference in bone volume and bone density between the BMSC/DBM in DM group (199 ± 63 mm^3^ and 593 ± 65 mg HA/ccm) and the BMSC/DBM in non-DM group (211 ± 39 mm^3^ and 608 ± 53 mg HA/ccm). Our data suggested that BMSC/DBM constructs could repair large bone defects in diabetic rats, but with delayed healing process compared with non-diabetic rats.

**Conclusion:**

Our study suggest that biomaterial sacffolds seeded with allogenic fetal BMSCs represent a promising strategy to induce and improve bone regeneration under diabetic condition.

## Introduction

Diabetes mellitus (DM) is a chronic metabolic disease characterized by the presence of elevated blood glucose levels, and is of two types: type 1 diabetes, which is due to severe deficiency in insulin synthesis, and type 2 diabetes which is caused by insulin resistance combined with insulin production deficiency (Antar et al., 2023). DM is a systematic disease affecting not only the heart, brain, kidneys, eyes, blood vessels and nerves, but it also causes a negative effect on the skeletal system. It is well known that DM contributes to osteoporosis, increased fracture risk, delayed bone healing and impaired bone regeneration (Antar et al., 2023; Csonka and Lendvay, 2023). In fact, it was reported that diabetic patients had a 6- to 7-fold increased risk of hip fracture, 2.75-fold increased risk of implant failure and 1.6-fold delay in fracture healing compared with non-diabetic population (Hu et al., 2018; Hygum et al., 2019). With the rise in incidence of DM and aging of population, the number of DM patients requiring bone reconstruction procedures rapidly increases. However, the repair of bone fracture and defect in diabetic patients still remains a major clinical challenge (Hu et al., 2018). Thus, it is essential to develop effective methods to accelerate bone regeneration in DM patients.

Bone tissue engineering has recently emerged as a promising treatment strategy for the repair and functional regeneration of large segmental bone defects (Li et al., 2018). Its general principle involves the integration of seed cells, tridimensional biomaterial framework and molecular signals to generate an implantable construct (Perez et al., 2018). This approach has been proved effective in both animal models and clinical trials (Confalonieri et al., 2018; Mazzoni et al., 2023). Nevertheless, most of the studies were performed in healthy recipients with unimpaired bone regeneration activity. There are limited studies on whether bone tissue engineering approach could improve bone regeneration under diabetic condition.

One of the fundamental components of bone tissue engineering strategy is seed cells. Bone marrow-derived mesenchymal stem cells (BMSCs) are a major source of osteoblasts, osteocytes and bone lining cells *in vivo*, and are crucial for bone remodeling via both cellular and paracrine effects. Because of their easy acquisition, potent proliferative and osteogenic potential, well-defined osteogenic differentiation pathway and low immunogenicity, BMSCs are considered as an attractive cellular source for bone tissue engineering applications (Zhang et al., 2012; Grelewski et al., 2023). Although autologous cell sources are generally preferred in clinical trials, it is well documented that BMSCs derived from diabetic patients exhibited decreased proliferative and osteogenic potential (Fijany et al., 2019). In addition, it was reported that diabetic donors possessed fewer BMSCs and that these cells exhibited impaired proliferation and survival *in vitro* (De Vyver, 2017). On the other hand, the use of allogenic BMSCs derived from healthy donors with efficacious osteogenic potential may circumvent the limitations of autologous BMSCs. Besides, allogenic BMSCs can be easily cultured, expanded and cryopreserved, allowing off-the-shelf availability. Investigations into their use in healing critical-sized bone defects in various animal models demonstrated their utility for bone tissue engineering applications (Berner et al., 2013; Arthur and Gronthos, 2020).

Decalcified bone matrix (DBM) is an artificial bone material obtained by decalcifying biological bone. It is considered as an ideal bone regeneration scaffold due to its good biocompatibility and osteogenic activity (Liu et al., 2023). Previously, we have conducted experiments to investigate the potential of allogenic fetal BMSC-based bone tissue engineering strategy for healing critical-sized bone defect in a rabbit model of osteoporosis (Wang et al., 2015). In the current study, we seeded allogenic fetal BMSCs on DBM scaffolds to construct tissue-engineered bone grafts, and investigated their potential to form both ectopic and orthotopic bone in streptozotocin (STZ)-induced type 1 diabetic rat model.

## Materials and methods

### Isolation and culture of BMSCs

To obtain fetal BMSCs, pregnant SD rats (19 days post conception) were sacrificed. Single-cell suspensions were prepared by flushing the bone marrow out of the femurs of rat fetuses using Dulbecco’s Modified Eagle’s Medium (DMEM; Hyclone, Logan City, UT, United States of America) containing 10% fetal bovine serum (FBS; Hyclone) under aseptic conditions. Cells were seeded in culture dish and cultured with low-glucose DMEM supplemented with 10% FBS and 1% penicillin-streptomycin antibiotic (Gibco, Grand Island, NY, United States of America) in a humidified incubator with 5% CO_2_ at 37°C. After 5 days, the culture medium was changed and non-adherent cells were removed. Cell passaging was performed until the monolayer of the adherent cells reached 80% confluence with trypsin-EDTA (Gibco).

### Multilineage differentiation of BMSCs

Osteogenic, adipogenic and chondrogenic differentiation of BMSCs was performed as previously described (Zhang et al., 2009). All chemicals were purchased from Sigma (St Louis, MO, United States of America) unless otherwise stated. For osteogenic differentiation, BMSCs at passage 3 were cultured in osteogenic induction medium (DMEM supplemented with 10% FBS, 1% penicillin-streptomycin antibiotic, 10 mmol/L β-glycerophosphate, 10^–8^ mol/L dexamethasone, and 50 mmol/L ascorbic acid). The medium was changed twice a week for 14 days. After induction, cells were fixed with 10% formalin and then stained with alizarin red.

For adipogenic differentiation, BMSCs at passage 3 were cultured in DMEM supplemented with 10% FBS, 1% penicillin-streptomycin antibiotic, 5 μg/mL insulin, 200 μM indomethacin, 1 μM dexamethasone, and 0.5 mM 3-isobutyl-1-methylxanthine. The medium was changed twice a week. After 21 days of induction, cells were fixed with 10% formalin and then stained with 0.5% oil red O in methanol.

For chondrogenic differentiation, BMSCs at passage 3 were pelleted and cultured in DMEM supplemented with 10% FBS, 1% penicillin-streptomycin antibiotic, 0.1 μM dexamethasone, 0.17 mM ascorbic acid, 1 mM sodium pyruvate, 0.35 mM L-proline, 1% insulin-transferrin sodium-selenite, 1.25 mg/mL bovine serum albumin, 5.33 μg/mL linoleic acid, and 0.01 μg/mL transforming growth factor-β (Cell Science, Canton, MA, United States). The medium was changed twice a week. After 28 days of incubation, the micromass pellets were fixed with 10% formalin, then embedded in paraffin and sectioned in 10-μm slices. After being dewaxed and rehydrated, the slices were stained with toluidine blue.

### Fabrication and characterization of BMSC/DBM construct

DBM scaffolds were prepared from bovine limbs as previously described (Wang et al., 2015). The scaffolds were cut into 10 mm × 5 mm × 3 mm cuboids, and then sterilized with 75% ethanol. BMSCs at passage 3 were harvested with EDTA-trypsin and resuspended with DMEM at a cellular density of 2 × 10^6^/mL. Each DBM cuboid was seeded with 100 μL cell suspension to generate BMSC/DBM construct. After 4 h of incubation in a humidified incubator at 37°C, the BMSC/DBM constructs were replenished with osteogenic induction medium and the medium was changed twice a week. After 14 days of osteogenic induction, BMSC/DBM constructs were ready for transplantation.

For scanning electron microscopic (SEM) analysis, the BMSC/DBM constructs were fixed in 2.5% glutaraldehyde after 14 days of osteogenic induction. The fixed BMSC/DBM constructs were then soaked in 1% osmic acid for 2 h and then rinsed with PBS. After being dehydrated with an ascending sequence of ethanol and dried at room temperature, the samples were sputter-coated with gold and finally observed with an SEM (Hitachi, Tokyo, Japan).

### Establishment of DM rat model

All animal protocols were approved by the University Committee on Use and Care of Animals of Huazhong University of Science and Technology. STZ is widely used to produce DM model in rats (Furman, 2021). As previously described with some modifications (Wojcicka et al., 2010), STZ (Sigma) was dissolved in 0.1 M of a citrate buffer (pH 4.5), and injected intraperitoneally into 5-week-old male Sprague-Dawley rats at a dose of 50 mg/kg body weight after overnight fasting. Rats in control group were injected with citrate buffer. Five days after STZ injection, fasting glucose (IFG) level in the blood obtained from the tail was measured using a glucometer (Omron, Kyoto, Japan). IFG >11.1 mmol/L and stable for 2 weeks were considered diabetes.

### *In vivo* study

To test the ability of BMSC/DBM constructs to form ectopic bone, twelve diabetic rats and six age-matched non-diabetic rats were divided into three group: 1) BMSC/DBM in non-DM group (n = 6): BMSC/DBM constructs were implanted subcutaneously into the dorsum of the non-diabetic control rats; 2) BMSC/DBM in DM group (n = 6): BMSC/DBM constructs were implanted subcutaneously into the dorsum of DM rats; 3) DBM in DM group (n = 6): cell-free DBM scaffolds were implanted subcutaneously into the dorsum of DM rats. After 1 and 3 months of implantation, the specimens were harvested and fixed in 4% formalin for further analyses.

To investigate the potential of BMSC/DBM constructs to repair large bone defects under diabetic condition, rat femoral large bone defect model was prepared as previously described (Janko et al., 2019). Twenty diabetic rats and ten age-matched non-diabetic rats were anaesthetized with isoflurane. The left hindlimb of the rat was shaved and cleaned with iodophor, and incision was made from lateral side of the left femur. Next, muscles and subcutaneous tissues were blunt-dissected to expose the left femoral shaft. An internal fixation plate was placed on the surface of the femoral shaft, and four screws fixed the plate to the bone. Afterwards, a 10-mm-long bone defect was created in the femur by an electrical bone saw. Then the rats were divided into three groups as mentioned above, the defects were filled with BMSC/DBM constructs or cell-free DBM scaffolds. The wound was closed and disinfected with iodophor. After the surgery, the rats were given 20,000 U/d gentamycin (Sigma) by intramuscular injection for 3 days. At the 6th month after the surgery, the rats were painlessly killed by overdosed inhalation of carbon dioxide. Femurs were harvested, and fixed in 4% formalin for further analyses.

### X-ray and micro-CT analyses

At the 1st, 3rd and 6th month after surgery, plain x-ray images were obtained under anesthesia with isoflurane. For micro-CT (μ-80, Scanco Medical, Zurich, Switzerland) analysis, bone specimens were placed in the sample holder and scanned at multiple longitudinal and transverse sections with 0.05 mm thickness. Then three-dimensional (3D) images were reconstructed. Bone volume (BV), bone volume per tissue volume (BV/TV) and bone density of the specimens was automatically calculated via micro-CT auxiliary software (Volume Graphics GmbH, Heidelberg, Germany).

### Histological evaluation

After micro-CT analysis, bone specimens were decalcified with 10% EDTA solution, dehydrated in increasing ethanol concentration, and then embedded in paraffin. Sections were cut into 10-μm-thick sections and stained with hematoxylin and eosin (HE, Sigma) and Van Gieson (VG, Sigma) to visualize tissue morphology and demonstrate collagen formation.

### Statistical analysis

All data collected are presented as mean ± standard deviation, and were analyzed using one-way ANOVA analysis. *p* < 0.05 was considered significant.

## Results

### Characterization of rat BMSCs and BMSC/DBM constructs

**FIGURE 1:**
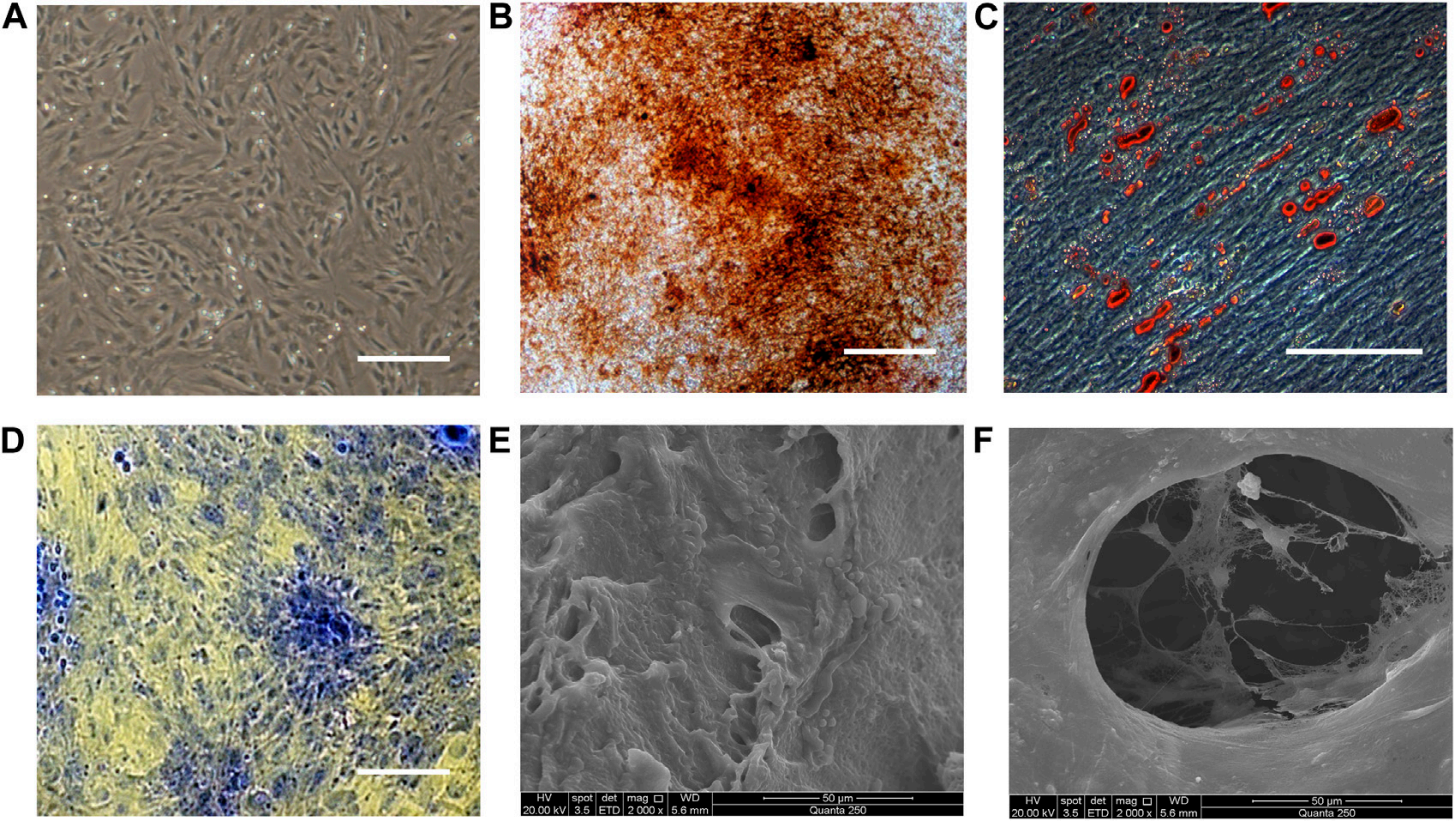
Characterization of BMSCs and BMSC/DBM constructs. **(A)**. Typical morphology of rat BMSCs at passage 3. Scale bar: 200 μm. **(B)**. Alizarin red staining of BMSCs after 14 days of osteogeneic induction. Scale bar: 100 μm. **(C)**. Oil red O staining of BMSCs after 21 days of adipogenic induction. Scale bar: 100 μm. **(D)**. Toluidine blue staining of the BMSC-derived micromass after 28 days of chondrogenic induction. Scale bar: 100 μm. **(E,F)**. BMSC/DBM constructs observed under SEM after 14 days of culture. Scale bars: 50 μm.

Rat fetal BMSCs exhibited a spindle-like morphology at passage 3 (Figure 1A). As shown in Figures 1B–D, MSCs underwent osteogenic, adipogenic, and chondrogenic differentiation, as demonstrated by alizarin red, oil red O and toluidine blue staining. The capacity of BMSCs to undergo multilineage differentiation was thereby established. To fabricate BMSC/DBM constructs, BMSCs were seeded on DBM scaffolds and then cultured in osteogenic induction medium for 14 days. SEM showed that after 14 days of culture and osteogenic induction, BMSCs on the DBM scaffolds grew vigorously, secreting abundant extracellular matrix and filling the pores of the scaffolds (Figures 1E, F).

### BMSC/DBM constructs form ectopic bone under diabetic condition

**FIGURE 2:**
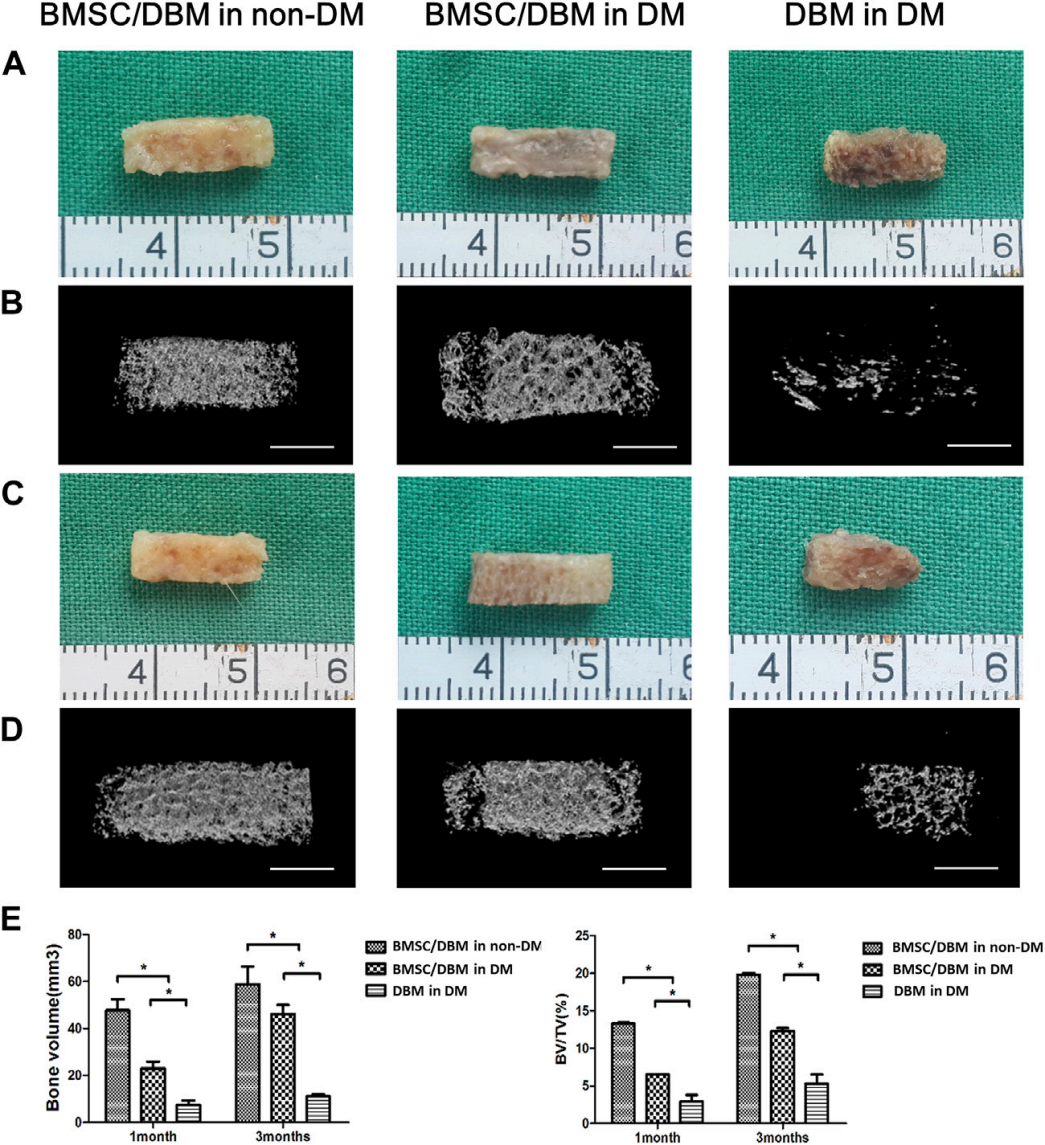
BMSC/DBM constructs form ectopic bone under diabetic condition. **(A)**. Macroscopic view of the specimens after 1 month of implantation. **(B)**. 3D reconstruction of micro-CT images of the specimens after 1 month of implantation. Scale bars: 5 mm. **(C)**. Macroscopic view of the specimens after 3 months of implantation. **(D)**. 3D reconstruction of micro-CT images of the specimens after 3 months of implantation. Scale bars: 5 mm. **(E)**. Statistical analyses of bone volume (BV) and bone volume per tissue volume (BV/TV), *p < 0.05.

To determine whether BMSC/DBM constructs could form ectopic bone under diabetic condition, we implanted BMSC/DBM constructs or cell-free DBM scaffolds subcutaneously into the dorsum of diabetic rats and age-matched non-diabetic rats. At 1 and 3 months after implantation, the specimens were harvested and scanned by micro-CT for evaluation. From the macroscopic view, both the BMSC/DBM in non-DM group and the BMSC/DBM in DM group showed complete tissue morphology, while scaffold degradation had already occurred in the DBM in DM group (Figures 2A, C). The 3D reconstruction of micro-CT images of the specimens showed that obvious mineralized tissue formation can be seen in both the BMSC/DBM in non-DM group and the BMSC/DBM in DM group, while the DBM in DM group showed hardly any mineralized tissue formation (Figures 2B, D). Quantitative micro-CT analysis (Figure 2E) showed that after 1 month of implantation, the BV and BV/TV of the BMSC/DBM in non-DM group (47.75 ± 4.8 mm^3^ and 13.3% ± 0.13%) were significantly increased compared with that of the BMSC/DBM in DM group (23.2 ± 2.9 mm^3^ and 6.5% ± 0.03%) and DBM in DM group (7.4 ± 1.73 mm^3^ and 2.9% ± 0.9%). After 3 months of implantation, the BV and BV/TV of the BMSC/DBM in non-DM group had increased to 58.9 ± 7.15 mm^3^ and 19.8% ± 0.25%, followed by the BMSC/DBM in DM group (46 ± 4.4 mm^3^ and 12.3% ± 0.41%) and DBM in DM group (11.1 ± 0.9 mm^3^ and 5.3% ± 1.2%). Our data demonstrated that BMSC/DBM constructs could form ectopic bone under diabetic condition, however, they showed impaired bone regeneration activity under diabetic condition compared with non-diabetic controls.

### BMSC/DBM constructs repair femoral bone defects under diabetic condition

**FIGURE 3:**
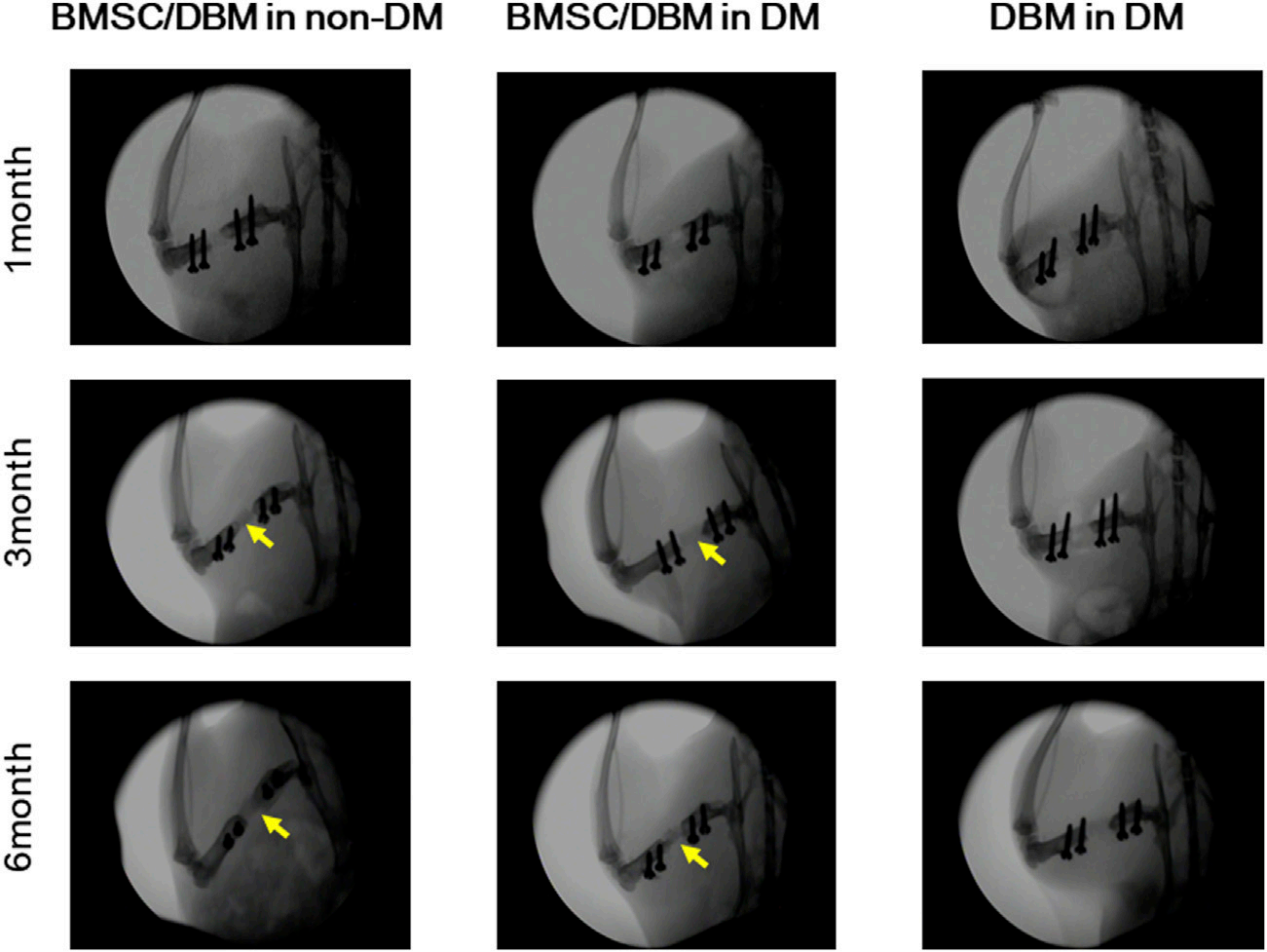
Plain X-ray images of the femoral defects at 1, 3 and 6 months after transplantation with BMSC/DBM constructs or DBM scaffolds in diabetic and non-diabetic rats.

Large bone defects were created in the femurs of both diabetic rats and age-matched non-diabetic rats. To investigate the potential of BMSC/DBM constructs to form orthotopic bone under diabetic condition, BMSC/DBM constructs or cell-free DBM scaffolds were implanted into the defects. X-ray examination showed that dense tissue was formed in both the BMSC/DBM in non-DM group and the BMSC/DBM in DM group at 3 months after surgery, and the BMSC/DBM in non-DM group showed much better new bone formation than the BMSC/DBM in DM group. At 6 months after surgery, bone union was observed in both the BMSC/DBM in non-DM group and the BMSC/DBM in DM group. In contrast, new bone formation did not occur in the DBM in DM group (Figure 3).

**FIGURE 4:**
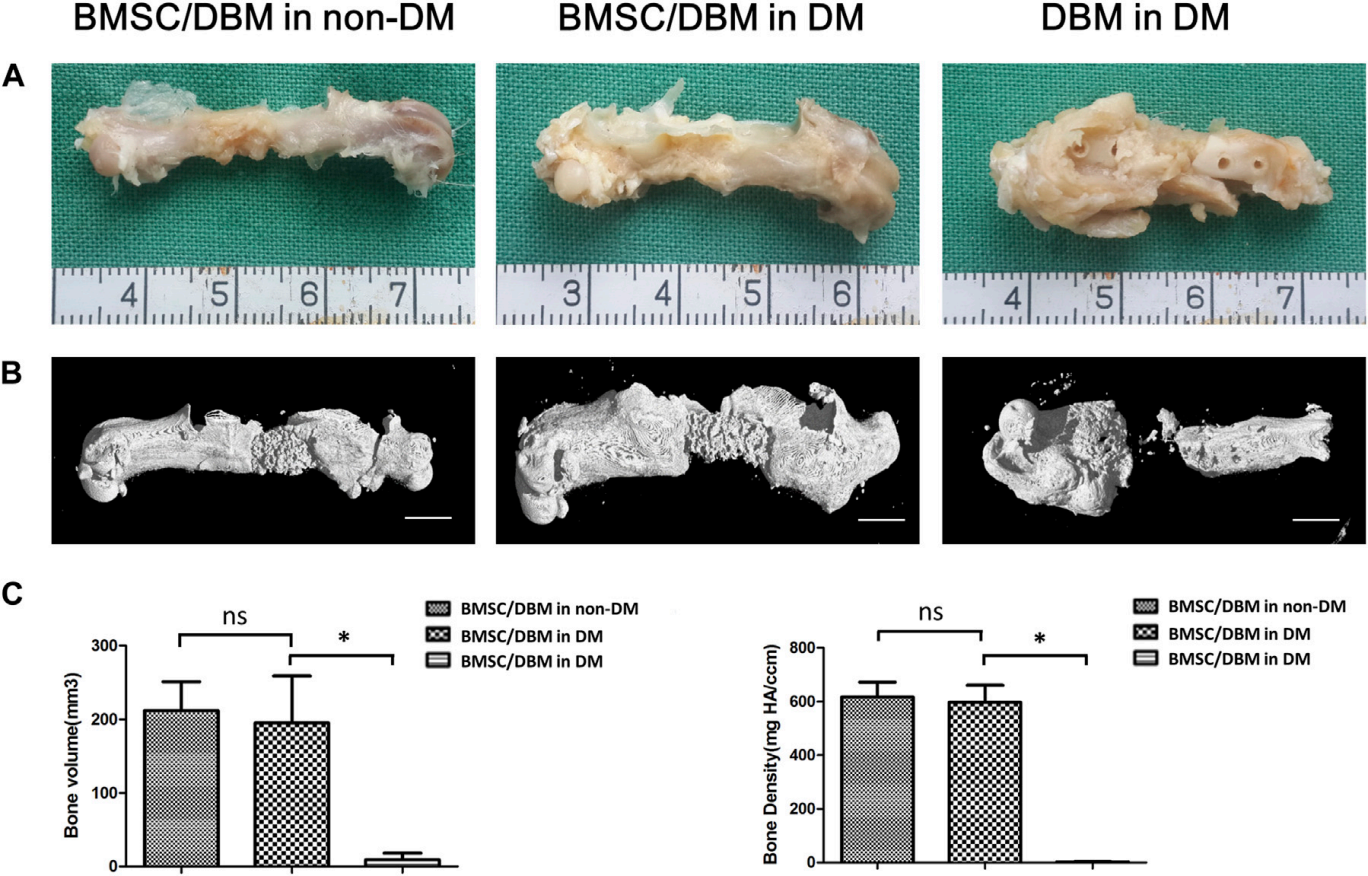
BMSC/DBM constructs repair femoral bone defects under diabetic condition. **(A)**. Macroscopic view of the specimens at the 6th month post-surgery. **(B)**. 3D reconstruction of micro-CT images of the specimens at the 6th month post-surgery. Scale bars: 5 mm. **(C)**. Statistical analyses of bone volume (BV) and bone density, *p < 0.05.

Animals were sacrificed at the 6th month post-surgery, femurs were obtained and then subjected to micro-CT analysis. As shown in Figures 4A, B, both macroscopic and 3D reconstructed micro-CT images showed that the defects in BMSC/DBM treated diabetic and non-diabetic group were filled with newly formed bone tissue. However, little new bone formation was observed in the DBM in DM group. Quantitative micro-CT analyses indicated that at 6 months after surgery, there was no significant difference in BV and bone density between the BMSC/DBM in non-DM group (211 ± 39 mm^3^ and 608 ± 53 mg HA/ccm) and BMSC/DBM in DM group (199 ± 63 mm^3^ and 593 ± 65 mg HA/ccm), although the BV and bone density were slightly higher in the non-diabetic group compared with the diabetic group (Figure 4C). These data suggest that BMSC/DBM constructs could repair large bone defects in diabetic rats, but with delayed healing process compared with non-diabetic rats.

### Histological analysis

**FIGURE 5:**
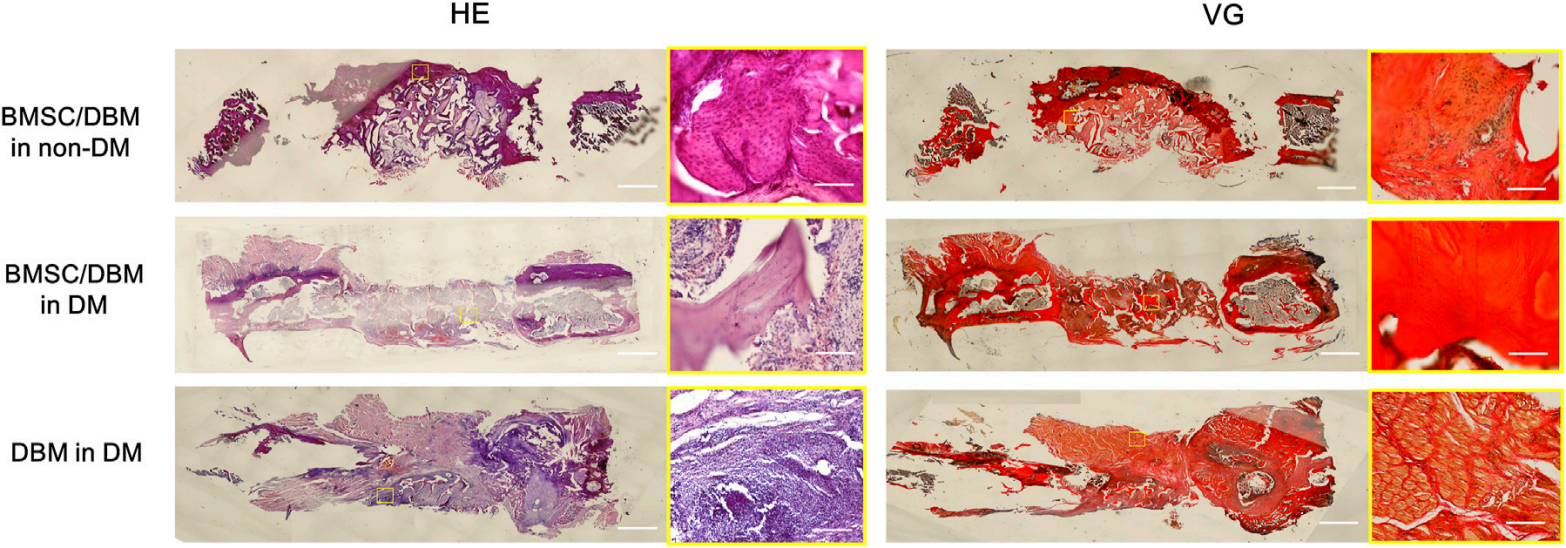
Histological analysis: representative images of HE and VG staining of the specimens. Scale bar: low magnifictaion: 2.5 mm; high magnification: 50 μm.

HE staining and Van Gieson staining were carried out to evaluate new bone formation. As shown in Figure 5, at 6 months post-surgery, newly formed bone tissue was observed in both the BMSC/DBM in non-DM and the BMSC/DBM in DM group. In contrast, the defect area in the DBM in DM group was filled with mostly fibrous connective tissue. Van Gieson staining further demonstrated that well-formed collagen deposition was observed in both the BMSC/DBM in non-DM and the BMSC/DBM in DM group, while hardly any collagen deposition can be seen in the DBM in DM group.

## Discussion

Diabetes is a group of metabolic disorders which can cause multiple organ damages. Patients with diabetes are more prone to osteoporosis, bone fracture and impaired bone healing than the non-diabetic population (Hofbauer et al., 2022). The rapidly expanding DM patients requiring bone reconstruction procedures motivated the present study to investigate the potential of bone tissue engineering approach to improve bone regeneration under diabetic condition. We implanted BMSC/DBM constructs or cell-free DBM scaffolds in both ectopic and orthotopic bone formation rat models. Our data suggested that BMSC/DBM constructs could form ectopic and orthotopic bone under diabetic condition, but with impaired bone regeneration activity.

It is well known that BMSCs derived from diabetic patients showed decreased alkaline phosphatase (ALP) activity, mineralization and osteogenic gene expression (Silva et al., 2015). It is also proposed that the balance of BMSC differentiation is switched to favor adipogenic differentiation under diabetic condition (Fijany et al., 2019). Besides, a number of studies suggested that the outcomes of autologous MSC therapy for diabetes-associated complications were variable and not as effective as initially hoped, due to the intrinsic MSC dysfunction (De Vyver, 2017). Therefore, autologous BMSCs may not be the ideal candidate to induce and improve bone regeneration under diabetic condition. Allogenic BMSCs have been shown to be nonimmunogenic in *vivo* transplantation paradigms (Squillaro et al., 2016), and have been used to repair critical-sized bone defects in various animal models (Lin et al., 2019), suggesting their utility for bone tissue engineering applications. In our study, no signs of serious adverse immune reactions were observed. Compared with perinatal and adult sources of MSCs, fetal BMSCs exhibited greater cell proliferative capability, osteogenic potential and lower immunogenicity as reported by previous studies (Zhang et al., 2009; Guillot et al., 2008), thus we chose allogenic fetal BMSCs as our seed cells in the present study.

Our data indicated that BMSC/DBM constructs could form ectopic bone under diabetic condition, but with decreased bone regeneration activity. Previous studies suggested that the osteogenic cells that formed new bone in subcutaneous ectopic bone formation models were mainly of donor origin rather than originated from the local microenvironment (Abdallah et al., 2008; Mankani et al., 2008). Deng et al. demonstrated that BMSCs derived from healthy donors treated with diabetic serum showed a remarkable decrease in osteogenic activity, suggesting that pathologic conditions like diabetes could alter the function of BMSCs derived from healthy donors (Deng et al., 2018). There have been a large number of studies trying to clarify the precise mechanism underlying the detrimental effect of diabetes on BMSCs. It is proved that many factors from the diabetic microenvironment could contribute to the dysfunction of BMSCs, including hyperglycemia, chronic inflammation and increased advanced glycation end-products (AGE) formation (Kume et al., 2005; Li et al., 2007; Yang et al., 2010; Wang et al., 2013; Ko et al., 2015; Qu et al., 2018; Xu and Zuo, 2021). We suggested that the allogenic BMSCs introduced through bone tissue engineering approach were affected by various factors from the diabetic host environment, resulting in the impaired bone regeneration activity. It is worthwhile to track and examine the function of the implanted BMSCs in the future.

Apart from testing the pro-bone regeneration potential of tissue-engineered bone constructs in subcutaneous ectopic bone formation models, we also investigated their potential to repair large segmental bone defects in rat femurs. To our surprise, at 6 months after surgery, BMSC/DBM constructs under diabetic condition showed comparable bone volume and density compared with the non-diabetic controls. However, X-ray analysis at the 1st and 3rd month post-surgery indicated that the healing process was delayed in the diabetic group. The implantation of BMSC-seeded DBM scaffolds enhanced bone formation in orthotopic defects compared with the implantation of unseeded DBM scaffolds, suggesting that the transplanted allogenic BMSCs were able to induce and promote osseous regeneration in orthotopic defect models. However, the delayed healing process compared with non-diabetic controls indicated that the implanted BMSCs were affected by the diabetic host environment, resulting in impaired osteogenic activity. Unlike ectopic bone formation models, previous studies have shown that new bone formation in orthotopic defect models is induced by both host and donor cells, and implantation of donor MSCs can cause recruitment of the host cells (Zhou et al., 2015; Westhauser et al., 2017; Kim et al., 2020). We assume that the implanted BMSCs were able to recruit endogenous stem/progenitor cells to the defect site, and these recruited local stem/progenitor cells together with the transplanted allogenic seed cells were able to induce bone regeneration, eventually leading to bone bridging at the defect site. Future studies could define the origin of the cells that form new bone by labeling the seed cells with fluorescent marker and investigate the interaction between the host and donor cells. The difference in the outcomes of ectopic bone formation model and orthotopic defect model in our study may be attributed to different local environment (ectopic and orthotopic) as well as different osteogenic process (calcification and new bone formation).

Strategies to promote bone regeneration include providing stem/progenitor cells, growth factors, nutrients, and synthetic materials to the defect site to induce tissue regeneration (Shui-Ling et al., 2018). The concept of bone tissue engineering involves the combination of stem/progenitor cells, tridimensional biomaterial scaffolds and growth factors. Although bone tissue engineering approach seems to hold great potential in improving bone regeneration under diabetic condition, the implanted exogenous seed cells would inevitably be affected by the host diabetic microenvironment. Previous studies on bone regeneration strategy under diabetic condition mainly focused on osteoblasts or osteoclasts by delivering pro-osteogenic factors (such as BMP2, VEGFA, FGF-9, IGF-1, Vitamin D, etc.) (Wang et al., 2010; Wu et al., 2013; De Santana and Trackman, 2015; Wallner et al., 2015) or anti-bone resorption factors like parathyroid hormone-related protein (Ardura et al., 2016). A recent study by Hu et al. highlighted the importance of modulating the diabetic microenvironment. They incorporated interleukin 4 into the nanofibrous heparin-modified gelatin microsphere to polarize proinflammatory M1 macrophages into an anti-inflammatory M2 phenotype that facilitate osteogenic differentiation and bone formation (Hu et al., 2018). Their research demonstrated that modulating the diabetic microenvironment played a crucial role in improving bone regeneration under diabetic condition. Future studies could devote to combining these pro-bone regeneration strategies like modulating the diabetic microenvironment with bone tissue engineering approach to accelerate bone regeneration under diabetic condition.

## Conclusion

In the present study, we demonstrated tissue-engineered bone constructs could form both ectopic and orthotopic bone in diabetic rats with impaired bone regeneration activity. Our research suggest that bone tissue engineering approach provides a promising way to accelerate bone regeneration under diabetic condition. In future studies, tissue-engineered cell/scaffold constructs could be combined with pro-osteogenic and immunomodulating factors to accelerate bone regeneration under diabetic condition.

## Data Availability

The raw data supporting the conclusion of this article will be made available by the authors, without undue reservation.
